# More is not always better: higher lymph node yield is not associated with improved survival in stage I–III colorectal cancer

**DOI:** 10.3389/fonc.2026.1900616

**Published:** 2026-08-19

**Authors:** Nina Schraps, Abdullah Al-Mubarak, Siwen Zhang, Gerrit Wolters-Eisfeld, Eleftherios D. Papazoglou, Anastasios D. Giannou, Jakob R. Izbicki, Thilo Hackert, Nathaniel Melling, Baris Mercanoglu

**Affiliations:** 1 Department of General, Visceral and Thoracic Surgery, University Medical Center Hamburg-Eppendorf, Hamburg, Germany; 2 Section of Molecular Immunology and Gastroenterology, I. Department of Medicine, University Medical Center Hamburg-Eppendorf, Hamburg, Germany; 3 Division of General Surgery, Department of Surgery, The University of British Columbia, Vancouver, BC, Canada; 4 Department of Urologic Sciences, The University of British Columbia, Vancouver, BC, Canada

**Keywords:** colorectal cancer, colorectal cancer outcomes, colorectal cancer surgery, CRC, lymph node yield, lymphadenectomy

## Abstract

**Background:**

Colorectal cancer (CRC) is among the most prevalent malignancies worldwide and remains the second leading cause of cancer-related death. Curative treatment approaches rely on R0 resection with adequate regional lymphadenectomy. Current international guidelines recommend a lymph node yield (LNY) of at least 12 lymph nodes to ensure accurate pathological staging and to guide decisions regarding adjuvant therapy. However, it remains controversial whether this historically established threshold is sufficient to optimize oncological outcomes. The aim of this study was to evaluate the association between operative LNY and clinicopathological characteristics, disease-free survival (DFS), and overall survival (OS) in patients undergoing curative resection for CRC.

**Methods:**

This retrospective study included 333 patients who underwent colorectal cancer resection at a high-volume center in Germany between 2016 and 2023. Only patients with Union for International Cancer Control (UICC) stage I–III disease who underwent curative-intent R0 resection were included. Patients were stratified into two groups according to LNY: <20 versus ≥20 retrieved lymph nodes.

**Results:**

LNY of ≥20 was associated with distinct tumor- and treatment-related characteristics, including advanced local tumor stage, microsatellite instability (MSI), colon cancer, right hemicolectomy, and absence of neoadjuvant treatment. No significant differences were observed between the groups regarding nodal stage, number of positive lymph nodes, lymphatic invasion, surgical approach, urgency of surgery, intraoperative blood loss, major postoperative morbidity, anastomotic leakage, chyle leak, reoperation or length of hospital stay. Higher LNY was not independently associated with improved OS or DFS in the overall cohort. Subgroup analyses stratified by UICC stage showed no significant association between LNY ≥20 on OS or DFS in stage I, II, or III disease. In procedure-specific analyses, LNY ≥20 was associated with improved OS after anterior rectal resection.

**Conclusions:**

Overall, these findings suggest that higher LNY is not a general prognostic determinant of oncological outcomes, but may have procedure-specific prognostic relevance.

## 1 Introduction

Colorectal cancer (CRC) is the third most commonly diagnosed cancer worldwide and the second leading cause of cancer-related death (1, 2). For patients with non-metastatic disease, curative treatment is based on complete oncologic resection with adequate regional lymphadenectomy (3–5). Accurate pathological staging, particularly the assessment of regional lymph node status, is critical for guiding adjuvant therapy decisions and predicting patient outcomes (6). International guidelines, including those of the American Joint Committee on Cancer (AJCC) and the National Comprehensive Cancer Network (NCCN), recommend examination of at least 12 lymph nodes after oncologic resection to ensure accurate nodal staging in CRC surgery (7–10). This benchmark has become an established quality indicator in colorectal cancer surgery and pathology, as retrieval of 12 or more lymph nodes has been associated with improved overall survival and reduced recurrence, likely reflecting more accurate staging and potentially more complete oncologic resection (11–13).

Extended lymphadenectomy beyond 12 lymph nodes has not consistently been associated with additional improvements in disease-free survival or overall survival in unselected patients (14–17). In contrast, some studies suggest that, in node-positive disease, a more extensive lymphadenectomy may reduce local recurrence and improve survival (15, 16, 18). Nevertheless, accumulating evidence indicates that the traditional 12-node benchmark may be insufficient to optimize oncologic outcomes. Several large population-based studies have demonstrated that higher lymph node yields (LNY) are independently associated with improved overall and disease-free survival across different stages of CRC (11, 19–21).

Although improved staging accuracy and reduced stage migration are plausible explanations, emerging translational research has challenged the traditional view of LNY as merely a surrogate marker of surgical and pathological quality (22–25). Despite the wealth of evidence, the optimal lymph node harvest target remains a matter of ongoing debate, with proposed thresholds ranging from 12 to 24 or more lymph nodes (26–28). This lack of consensus highlights the need for a nuanced, evidence-based re-evaluation of current standards for lymph node assessment in CRC.

The present study aimed to evaluate the association between LNY and clinicopathological characteristics, postoperative outcomes, disease-free survival (DFS), and overall survival (OS) in a cohort of patients undergoing curative resection for stage I–III colorectal cancer. In particular, we examined whether retrieval of 20 or more lymph nodes was associated with improved oncologic outcomes. The cutoff of 20 lymph nodes was selected on the basis of prior studies that evaluated higher LNY thresholds, typically ranging from 18 to 24 lymph nodes, in relation to overall survival and/or disease-free survival, with conflicting results (19–21, 29–32). Against this background, a cutoff of 20 lymph nodes was selected as a clinically interpretable, literature-based, and cohort-informed threshold to evaluate the potential prognostic relevance of a higher LNY.

## 2 Materials and methods

### 2.1 Study design and patient selection

**Figure 1:**
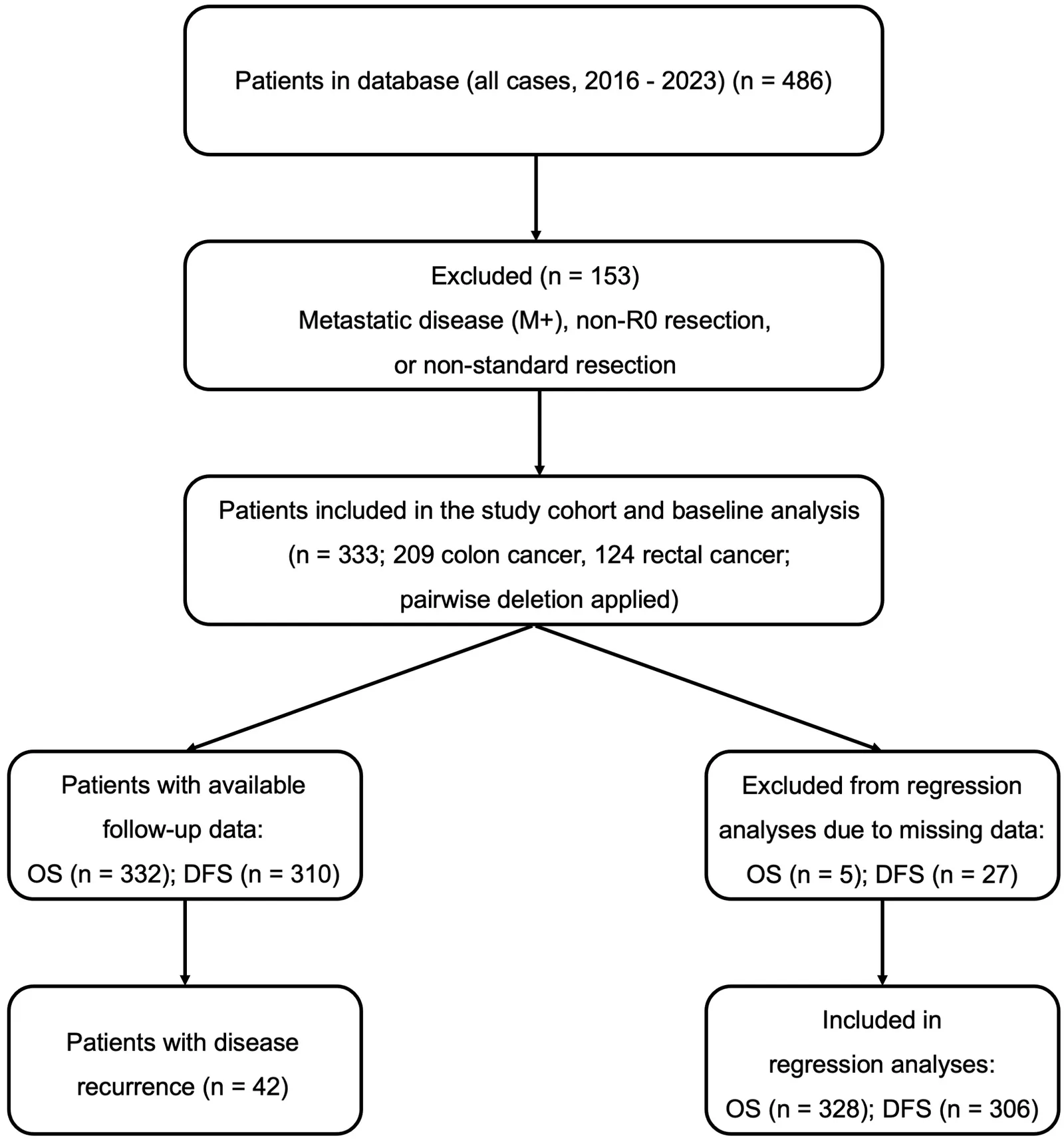
Flow diagram of patient selection.

This study was a retrospective analysis of a prospectively maintained database including consecutive patients who underwent colorectal surgery for newly diagnosed colorectal adenocarcinoma (UICC stage I–III) between January 2016 and December 2023 at the Department of General, Visceral and Thoracic Surgery, University Medical Center Hamburg-Eppendorf (UKE), Germany. A total of 486 patients were identified in the database. Of these, 153 were excluded because of metastatic disease (M+), non-R0 resection, or non-standard resection. The final study cohort comprised 333 patients. The patient selection flowchart is shown in **Figure 1**.

All surgical procedures were performed by board-certified colorectal surgeons at a colorectal cancer center certified by the German Cancer Society (Deutsche Krebsgesellschaft, DKG). Both elective and emergency procedures were included. Emergency surgery was defined as an operation performed within 6 hours after the indication for surgery had been established. Open, laparoscopic, and robot-assisted approaches were considered. All patients underwent standard oncologic colorectal resection with regional lymphadenectomy according to tumor location and multidisciplinary treatment planning.

Pathological examination was performed according to institutional standards by experienced gastrointestinal pathologists. This included macroscopic assessment of the resection specimen, manual lymph node dissection, histopathological evaluation, and documentation of the total number of retrieved and tumor-positive lymph nodes. Macroscopically identified lymph nodes were sectioned at 0.2-cm intervals and entirely submitted for histopathological work-up. For each lymph node, serial step sections with a thickness of 2 - 4 µm were prepared, resulting in at least four levels per lymph node. Microsatellite instability (MSI) status was assessed by immunohistochemistry for the mismatch repair (MMR) proteins MLH1, PMS2, MSH2, and MSH6. In cases with loss of MMR protein expression or equivocal staining patterns, additional PCR-based MSI testing was performed where appropriate.

Lymph node yield was defined as the total number of regional lymph nodes retrieved and examined in the resection specimen. Patients were stratified into two groups according to LNY: <20 versus ≥20 lymph nodes.

The clinical database was approved by the Ethics Committee of the City of Hamburg, and written informed consent was obtained from all patients prior to inclusion in the database. The study was conducted in accordance with the Declaration of Helsinki. The reporting of this study was performed in accordance with the Strengthening the Reporting of Observational Studies in Epidemiology (STROBE) guidelines (33). The primary outcome was overall survival. Secondary outcomes included disease-free survival and postoperative complications. In addition, patient, operative, and tumor characteristics were analyzed. Subgroup analyses were performed according to UICC stage I, II, and III. Furthermore, right hemicolectomy, left hemicolectomy, and anterior rectal resection were analyzed as prespecified procedure-specific subgroups.

### 2.2 Data collection

Clinical data were collected and managed using REDCap (Research Electronic Data Capture) electronic data capture tools hosted at University Medical Center Hamburg-Eppendorf, Germany (34, 35). REDCap is a secure, web-based software platform designed to support data capture for research studies.

The preoperative Charlson Comorbidity Index (CCI) was assessed before surgery (36). Postoperative complications were classified according to the Clavien-Dindo classification system (37). Preoperative diagnostic workup was performed in accordance with the German S3 guideline for colorectal carcinoma (38). Overall survival was defined as the time from the date of surgery to death from any cause, as recorded in the German National Population Register, or to the date of last contact with the population register (last follow-up). Disease-free survival was defined as the time from the date of surgery to the first documented local or distant disease recurrence. The date of recurrence was defined as the earliest date of radiological or pathological evidence of relapse. Patients without an event were censored at the date of the last documented disease assessment or last clinical follow-up. Recurrence status was determined by systematic chart review, including external medical reports transmitted to our center. Only radiologically and/or pathologically confirmed recurrences were considered.

### 2.3 Statistical analysis

Statistical analyses were performed using R version 4.3.0 (R Core Team, 2023) with the survival package. The distribution of continuous variables was assessed using a combination of the Shapiro-Wilk test and graphical methods, including histograms, Q-Q plots, and boxplots. Continuous variables with non-normal distributions and ordinal variables were summarized as medians with interquartile ranges (IQR), whereas normally distributed continuous variables were summarized as means with standard deviations (SD). Categorical variables were reported as absolute and relative frequencies. Univariable associations between categorical variables were assessed using the chi-square test or Fisher exact test, as appropriate, and continuous variables were compared using the Mann-Whitney U test. For the comparison of baseline characteristics between the two LNY groups, pairwise deletion was applied to variables with missing data. Overall survival and disease-free survival were estimated using the Kaplan-Meier method, and survival differences between groups were compared with the log-rank test.

Multivariable Cox proportional hazards regression models were used to identify independent prognostic factors and to account for potential confounding by clinically relevant baseline variables. Clinically relevant baseline variables with established or plausible prognostic relevance for overall survival and disease-free survival after colorectal cancer surgery were predefined as candidate variables. Univariable Cox regression analyses were then performed as a variable-reduction step. Variables with *p* < 0.05 in the univariable analysis were subsequently entered into the multivariable Cox proportional hazards model. This approach was used to construct a parsimonious model and to limit the number of covariates in relation to the number of observed outcome events, thereby reducing the risk of overfitting. In addition, multicollinearity among variables was assessed using variance inflation factors (VIFs), and no relevant collinearity was detected. The proportional hazards assumption was assessed using Schoenfeld residuals for each covariate and for the model globally. In the event of nonproportional hazards, the Cox model was stratified by the respective variable. For the regression analyses, a complete-case approach was used.

Overall significance of variables was assessed using likelihood ratio tests, and hazard ratios (HR) with 95% confidence intervals (CI) were reported. A two-sided *p*-value < 0.05 was considered statistically significant.

## 3 Results

### 3.1 Patient flow and study cohort

A total of 486 patients were identified in the database. Of these, 153 were excluded because of metastatic disease (M+), non-R0 resection, or non-standard resection. The final study cohort comprised 333 patients, including 209 with colon cancer and 124 with rectal cancer. The patient selection flowchart is shown in **Figure 1**.

The median LNY in the overall cohort was 20 lymph nodes. Patients were stratified into two groups according to LNY: <20 versus ≥20 lymph nodes. The <20 group comprised 163 patients, whereas the ≥20 group comprised 170 patients. All 333 patients were included in the baseline analyses presented in **Table 1**.

**Table 1:** Association between lymph node yield and clinicopathological characteristics in the overall cohort.

Characteristics	All patients (n=333)	LNY <20 (n=163)	LNY ≥20 (n=170)	*p*-value
**Age (years [IQR])**	66.00 [55.00, 75.00]	67.00 [58.00, 77.50]	64.50 [55.00, 75.00]	0.145
Gender [n (%)]				
Female	132 (39.6)	64 (39.3)	68 (40.0)	0.980
Male	201 (60.4)	99 (60.7)	102 (60.0)	
**BMI (kg/m^2^ [IQR])**	24.68 [21.80, 28.09]	24.91 [21.81, 28.44]	24.64 [21.76, 28.01]	0.599
**Charlson Comorbidity Index (SD)**	3.50 (2.61)	3.90 (2.81)	3.12 (2.35)	**0.006**
ECOG [n (%)]				
0	257 (77.4)	117 (72.2)	140 (82.4)	**0.013**
1	46 (13.9)	24 (14.8)	22 (12.9)	
2	17 (5.1)	10 (6.2)	7 (4.1)	
>2	12 (3.6)	11 (6.8)	1 (0.6)	
ASA [n (%)]				
I	10 (3.1)	3 (1.9)	7 (4.2)	0.078
II	133 (40.9)	57 (36.3)	76 (45.2)	
III	153 (47.1)	78 (49.7)	75 (44.6)	
IV	29 (8.9)	19 (12.1)	10 (6.0)	
Urgency of surgery [n (%)]				
Elective	289 (86.8)	145 (89.0)	144 (84.7)	0.325
Emergency	44 (13.2)	18 (11.0)	26 (15.3)	
Surgical approach [n (%)]				
Open	118 (35.4)	53 (32.5)	65 (38.2)	0.205
Laparoscopic	98 (29.4)	45 (27.6)	53 (31.2)	
Robotic	117 (35.1)	65 (39.9)	52 (30.6)	
Conversion [n (%)]				
No	303 (91.0)	147 (90.2)	156 (91.8)	0.755
Yes	30 (9.0)	16 (9.8)	14 (8.2)	
Tumor Location [n (%)]				
Colon	209 (62.8)	84 (51.5)	125 (73.5)	**<0.001**
Rectum	124 (37.2)	79 (48.5)	45 (26.5)	
Procedure [n (%)]				
Anterior rectum resection	104 (31.2)	67 (41.1)	37 (21.8)	**<0.001**
Left hemicolectomy	82 (24.6)	38 (23.3)	44 (25.9)	
Rectum extirpation	20 (6.0)	12 (7.4)	8 (4.7)	
Right hemicolectomy	127 (38.1)	46 (28.2)	81 (47.6)	
**Operative Time** **(minutes [IQR])**	229.00 [174.00, 274.00]	235.00 [179.50, 303.50]	216.00 [169.25, 265.75]	**0.034**
**Intraoperative blood loss** **[ml (SD)]**	287.16 (415.18)	264.58 (399.71)	309.51 (430.74)	0.442
**CEA [IQR]**	2.20 [1.10, 5.50]	2.10 [1.10, 4.00]	2.30 [1.20, 6.90]	0.354
UICC [n (%)]				
0	4 (1.2)	2 (1.2)	2 (1.2)	0.105
I	101 (30.3)	57 (35.0)	44 (25.9)	
II	117 (35.1)	47 (28.8)	70 (41.2)	
III	111 (33.3)	57 (35.0)	54 (31.8)	
Grading [n (%)]				
G1	10 (3.4)	6 (4.4)	4 (2.6)	0.159
G2	249 (85.3)	119 (86.9)	130 (83.9)	
G3	21 (7.2)	10 (7.3)	11 (7.1)	
G4	12 (4.1)	2 (1.5)	10 (6.5)	
pT [n (%)]				
ypT0 (pCR)	4 (1.2)	2 (1.2)	2 (1.2)	**0.035**
T1	44 (13.2)	26 (16.0)	18 (10.6)	
T2	78 (23.4)	46 (28.2)	32 (18.8)	
T3	169 (50.8)	77 (47.2)	92 (54.1)	
T4	38 (11.4)	12 (7.4)	26 (15.3)	
pN [n (%)]				
N0	222 (66.7)	106 (65.0)	116 (68.2)	0.586
N1	87 (26.1)	46 (28.2)	41 (24.1)	
N2a	17 (5.1)	9 (5.5)	8 (4.7)	
N2b	7 (2.1)	2 (1.2)	5 (2.9)	
pM [n (%)]				
M0	333 (100.0)	163 (100.0)	170 (100.0)	n.a.
Resection status [n (%)]				
R0	333 (100.0)	163 (100.0)	170 (100.0)	n.a.
**Positive Lymph nodes (SD)**	0.82 (1.90)	0.73 (1.40)	0.91 (2.28)	0.399
L-status [n (%)]				
L0	230 (69.9)	118 (73.3)	112 (66.7)	0.234
L1	99 (30.1)	43 (26.7)	56 (33.3)	
V-status [n (%)]				
V0	305 (92.7)	151 (93.8)	154 (91.7)	0.598
V1	24 (7.3)	10 (6.2)	14 (8.3)	
Pn-status [n (%)]				
Pn0	279 (91.5)	136 (90.7)	143 (92.3)	0.770
Pn1	26 (8.5)	14 (9.3)	12 (7.7)	
MSS/MSI [n (%)]				
MSI	49 (16.4)	14 (9.8)	35 (22.6)	**0.005**
MSS	249 (83.6)	129 (90.2)	120 (77.4)	
MERCURY [n (%)]				
I	123 (97.6)	73 (96.1)	50 (100.0)	0.410
II	3 (2.4)	3 (3.9)	0 (0.0)	
West [n (%)]				
I	97 (90.7)	37 (88.1)	60 (92.3)	0.206
II	8 (7.5)	5 (11.9)	3 (4.6)	
III	2 (1.9)	0 (0.0)	2 (3.1)	
Clavien-Dindo classification [n (%)]				
None	171 (51.4)	84 (51.5)	87 (51.2)	0.172
I	29 (8.7)	14 (8.6)	15 (8.8)	
II	46 (13.8)	16 (9.8)	30 (17.6)	
III a	15 (4.5)	10 (6.1)	5 (2.9)	
III b	34 (10.2)	16 (9.8)	18 (10.6)	
IV a	16 (4.8)	8 (4.9)	8 (4.7)	
IV b	3 (0.9)	3 (1.8)	0 (0.0)	
V	19 (5.7)	12 (7.4)	7 (4.1)	
Anastomotic leakage [n (%)]				
None	296 (88.9)	142 (87.1)	154 (90.6)	0.475
A	1 (0.3)	0 (0.0)	1 (0.6)	
B	13 (3.9)	7 (4.3)	6 (3.5)	
C	23 (6.9)	14 (8.6)	9 (5.3)	
Chyle leak [n (%)]				
No	329 (98.8)	162 (99.4)	167 (98.2)	0.645
Yes	4 (1.2)	1 (0.6)	3 (1.8)	
Reoperation for postoperative bleeding [n (%)]				
No	323 (97.0)	157 (96.3)	166 (97.6)	0.698
Yes	10 (3.0)	6 (3.7)	4 (2.4)	
**Length of hospital stay (days [IQR])**	9.00 [7.00, 15.00]	9.00 [7.00, 15.00]	10.00 [7.00, 15.00]	0.510
Neoadjuvant chemotherapy [n (%)]				
No	299 (89.8)	138 (84.7)	161 (94.7)	**0.004**
Yes	34 (10.2)	25 (15.3)	9 (5.3)	
Neoadjuvant radiotherapy [n (%)]				
No	294 (88.3)	136 (83.4)	158 (92.9)	**0.012**
Yes	39 (11.7)	27 (16.6)	12 (7.1)	
Adjuvant chemotherapy [n (%)]				
No	202 (60.8)	106 (65.0)	96 (56.8)	0.155
Yes	130 (39.2)	57 (35.0)	73 (43.2)	
Adjuvant radiotherapy [n (%)]				
No	321 (96.7)	154 (94.5)	167 (98.8)	0.057
Yes	11 (3.3)	9 (5.5)	2 (1.2)	

Data are presented as median (interquartile range [IQR]), mean (standard deviation [SD]), or n (%). Bold type indicates *p*-values < 0.05. Pairwise deletion was applied to variables with missing data. Right- and left-sided colon cancers are represented by the procedure-specific categories, with right hemicolectomy corresponding to right-sided colon cancer and left hemicolectomy corresponding to left-sided colon cancer.

n.a., not applicable, as patients with metastatic disease (M+) or non-R0 resection were excluded; ASA, American Society of Anesthesiologists; BMI, body mass index; ECOG, Eastern Cooperative Oncology Group; pCR, pathological complete response; UICC, Union for International Cancer Control.

Follow-up data were available for OS in 332 patients and for DFS in 310 patients. The median follow-up duration was 49.6 months (95% CI, 47.7 - 52.1; **Supplementary Table 1**) for OS and 45.9 months (95% CI, 41.2 - 49.6; **Supplementary Table 1**) for DFS. Overall, 42 patients experienced disease recurrence.

For the regression analyses, a complete-case approach was used, resulting in the inclusion of 328 of 333 patients (98.5%) in the OS model and 306 of 333 patients (91.9%) in the DFS model.

In the initial OS model, the proportional hazards assumption was violated for the Charlson Comorbidity Index (*p* = 0.002), and the global test was significant (*p* = 0.014). After stratification by the Charlson Comorbidity Index, no significant violation was detected for the remaining covariates or the overall model (global *p* = 0.275).

For the DFS model, both the covariate-specific tests and the global test were nonsignificant (global *p* = 0.978), indicating no violation of the proportional hazards assumption.

### 3.2 Associations of lymph node yield and characteristics of the patient cohort

**Table 1** shows the relationship between LNY and clinical as well as histopathological characteristics of the patient cohort. A total of 333 patients with CRC UICC stage I–III who underwent curative-intent R0 resection were included in this study. 170 patients had LNY ≥20, while 163 patients had LNY <20. With regard to the general health status of the patients, LNY ≥20 was more frequently observed in patients with a lower ECOG (Eastern Cooperative Oncology Group) performance status (*p* = 0.013) and Charlson Comorbidity Index (*p* = 0.006).

The primary tumor was located in the colon in 209 (62.8%) patients, including 127 (60.8%) right-sided colon cancers and 82 (39.2%) left-sided colon cancers. 124 (37.2%) patients had rectal cancer. LNY ≥20 was significantly associated with the tumor location and was more frequently found in patients with colon cancer (*p* < 0.001). Patients with LNY ≥20 more frequently presented with advanced local tumor stages (*p* = 0.035), mainly reflected by a higher proportion of pT3 and pT4 tumors and lower proportions of pT1 and pT2 tumors. In addition, microsatellite instability was more commonly observed in patients with LNY ≥20 (*p* = 0.005).

Neoadjuvant chemotherapy (*p* = 0.004) as well as neoadjuvant radiotherapy (*p* = 0.012) were significantly associated with LNY <20. No discrepancies were found in the frequency of adjuvant chemotherapy (*p* = 0.155) or radiotherapy (*p* = 0.057) between LNY <20 and ≥20. The type of surgical procedure differed significantly between the LNY groups (*p* < 0.001), particularly with a higher incidence of right hemicolectomy and a lower incidence of anterior rectal resection in patients with LNY ≥ 20. Operative time was shorter in patients with a LNY ≥20 (*p* = 0.034). No significant differences were found between the LNY groups regarding surgical approach, conversion rate, urgency of surgery, intraoperative blood loss, postoperative complications according to the Clavien-Dindo Classification, anastomotic leakage, chyle leak, reoperation rate or length of hospital stay (*p* ≥ 0.05).

There were no significant differences between the two groups regarding age, sex, body mass index (BMI), preoperative CEA levels, UICC stage, tumor grading, nodal stage, number of positive lymph nodes, lymphatic invasion, venous invasion, perineural invasion, MERCURY (Magnetic Resonance Imaging and Rectal Cancer European Equivalence) classification or West classification (*p* ≥ 0.05).

### 3.3 Lymph node yield and patient survival

**Figure 2:**
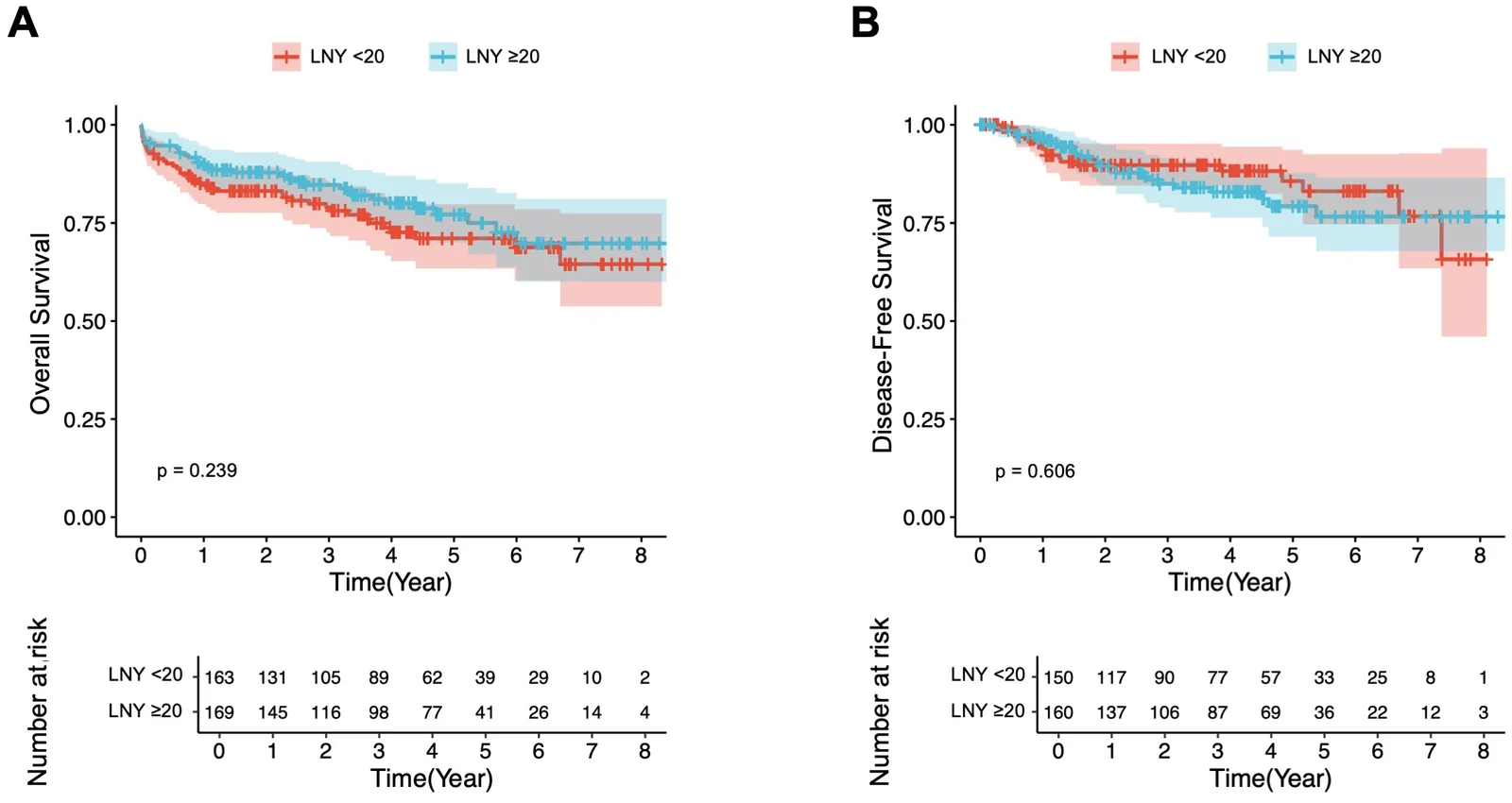
Kaplan-Meier survival curves showing the association of lymph node yield with overall survival **(A)** and disease-free survival **(B)** in the overall cohort.

**Figure 3:**
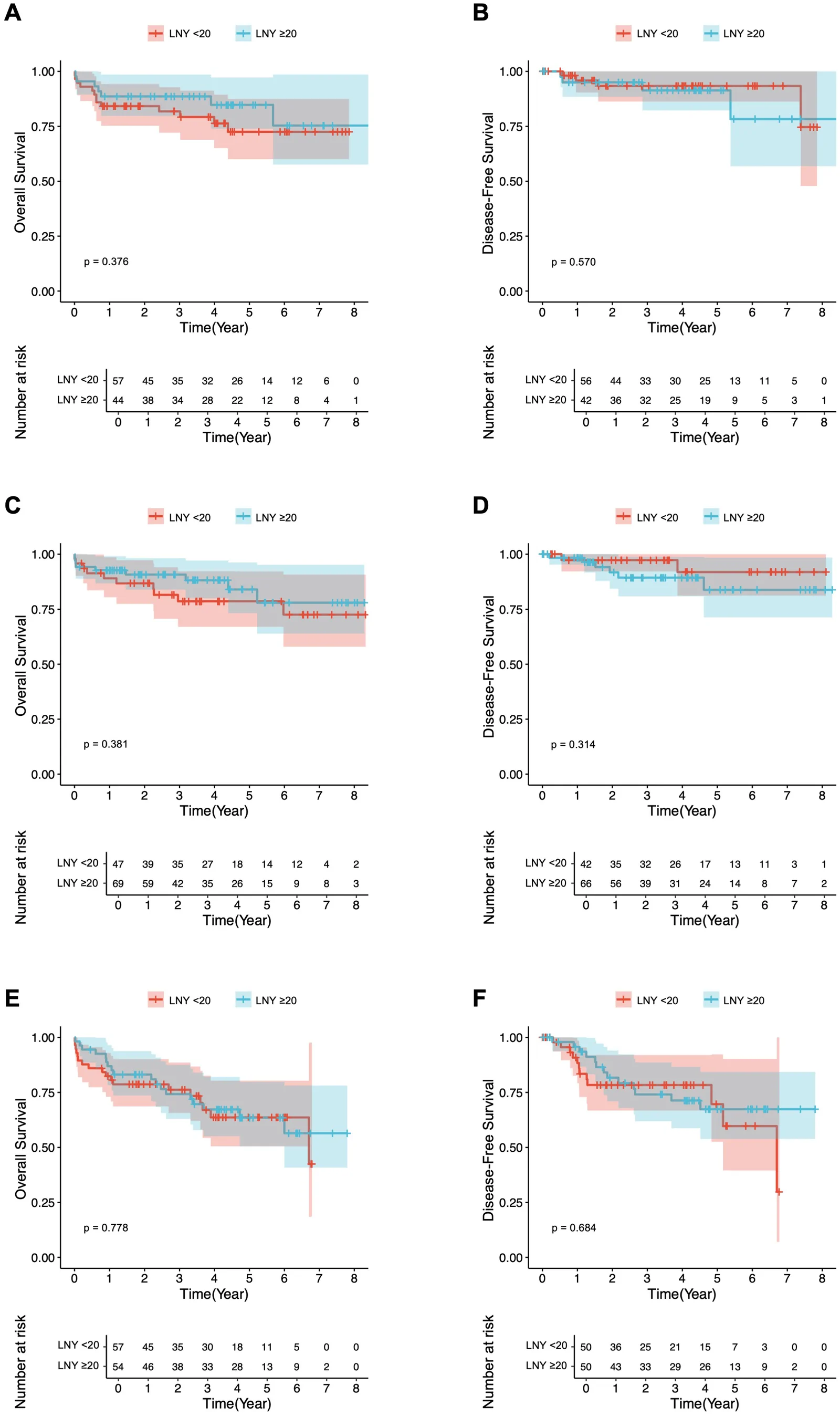
Kaplan-Meier survival curves illustrating the association between lymph node yield and overall survival **(A, C, E)** and disease-free survival **(B, D, F)** according to UICC stage I **(A, B)**, stage II **(C, D)**, and stage III **(E, F)**.

In the overall cohort, Kaplan-Meier analyses showed no significant association between lymph node yield and overall survival (*p* = 0.239; **Figure 2A**) or disease-free survival (*p* = 0.606; **Figure 2B**). This finding remained consistent after stratification by UICC stage, with no significant association between lymph node yield and either OS or DFS in patients with stage I, II, or III disease (**Figures 3A–F**; all *p* ≥ 0.05).

**Figure 4:**
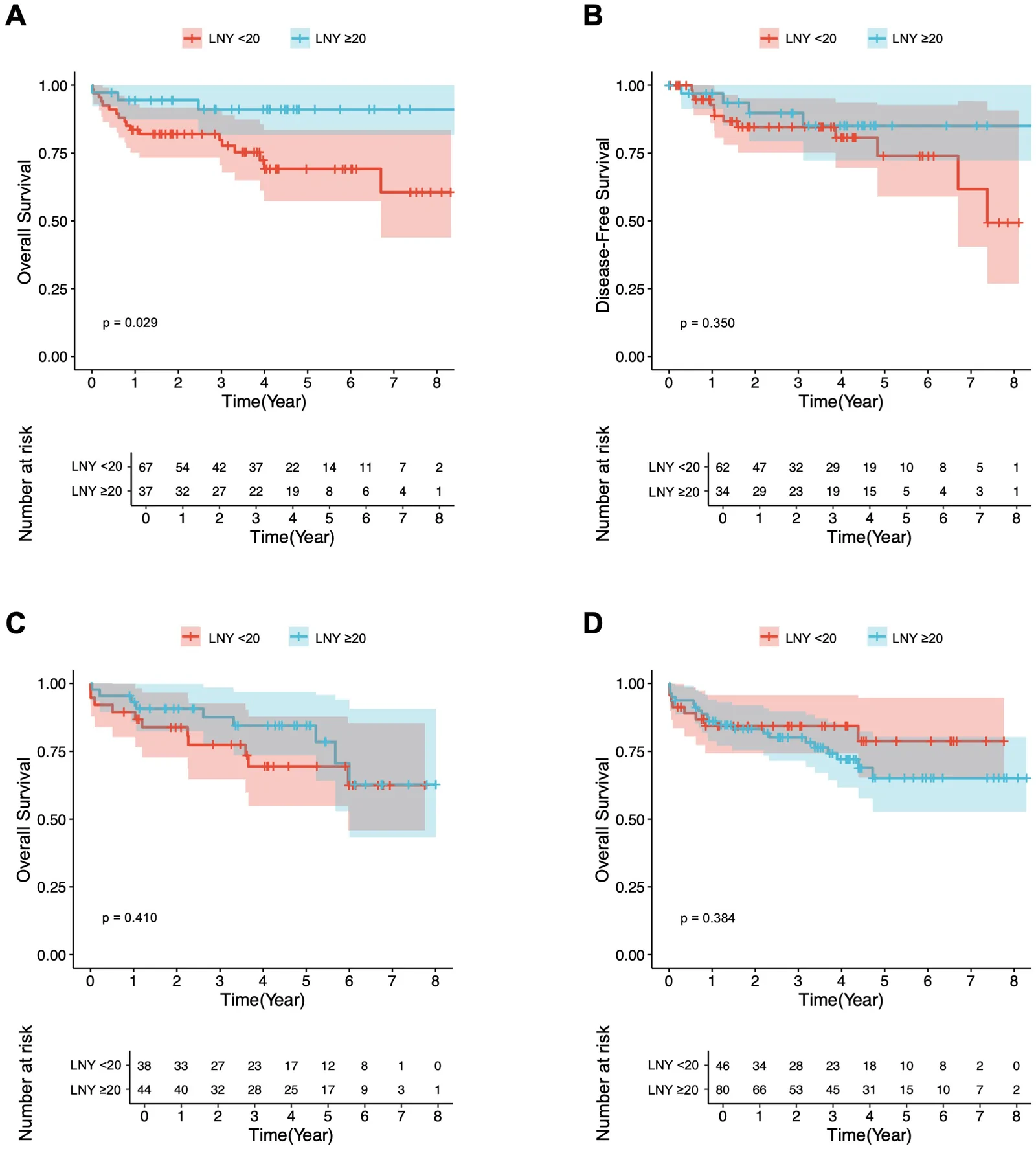
Kaplan-Meier survival curves showing the association between lymph node yield and overall survival **(A)** and disease-free survival **(B)** in patients undergoing anterior rectal resection. Overall survival is shown for left hemicolectomy **(C)** and right hemicolectomy **(D)**.

Procedure-specific exploratory analyses were performed for anterior rectal resection, left hemicolectomy, and right hemicolectomy. Among patients undergoing anterior rectal resection, LNY of ≥20 was associated with improved overall survival compared with LNY of <20 (*p* = 0.029; **Figure 4A**). However, no significant difference in disease-free survival was observed (*p* = 0.350; **Figure 4B**). In contrast, LNY of ≥20 was not associated with improved overall survival after left hemicolectomy (*p* = 0.410; **Figure 4C**) or right hemicolectomy (*p* = 0.384; **Figure 4D**).

Exploratory subgroup analyses were performed according to lymphatic invasion status. Among patients without lymphatic invasion, LNY of ≥20 was not associated with improved overall survival (*p* = 0.266; **Supplementary Figure 1A**) or disease-free survival (*p* = 0.642; **Supplementary Figure 1B**). This pattern was consistent among patients with lymphatic invasion, in whom no significant differences were observed between the LNY groups for overall survival (*p* = 0.531; **Supplementary Figure 1C**) or disease-free survival (*p* = 1.000; **Supplementary Figure 1D**).

### 3.4 Univariable and multivariable regression analyses for OS

The findings of the univariable and multivariable Cox regression analyses for overall survival are shown in **Table 2**. Consistent with the Kaplan-Meier analysis, higher LNY was not associated with OS (*p* = 0.241). When analyzed as a continuous variable, LNY was also not significantly associated with OS (HR per additional lymph node, 0.98; 95% CI, 0.96 - 1.01; *p* = 0.175). Lymphatic invasion (*p* = 0.145) and venous invasion (*p* = 0.114) were also not associated with OS.

**Table 2:** Univariable and multivariable Cox regression analyses of overall survival.

Characteristics	All patients (n=328)	Events (n=75)		Univariate analysis		Multivariate analysis	
			Restricted mean survival time in months (SE)	Hazard ratio (95% CI)	*P*-value	Hazard ratio (95% CI)	*P*-value
Age (%)							
<50	54 (16.5)	7	92 (4.66)				
≥50	274 (83.5)	68	79 (2.72)	2.13 (0.98 - 4.63)	0.057		
Gender (%)							
Female	129 (39.3)	25	85 (3.61)				
Male	199 (60.7)	50	79 (3.21)	1.36 (0.84 - 2.2)	0.208		
Charlson comorbidity index (%)							
<3	121 (36.9)	11	95 (2.78)				
≥3	207 (63.1)	64	73 (3.32)	4.09 (2.15 - 7.75)	**<0.001**	**Stratification variable**	
Urgency of surgery (%)							
Elective	285 (86.9)	61	83 (2.53)				0.309
Emergency	43 (13.1)	14	71 (7.45)	1.84 (1.03 - 3.3)	**0.04**	1.41 (0.74 - 2.7)	
Surgical approach (%)							
Open	116 (35.4)	38	70 (4.56)				0.122
Laparoscopic	96 (29.3)	22	83(4.08)	0.58 (0.34 - 0.98)	**0.041**	0.65 (0.37 - 1.15)	
Robotic	116 (35.4)	15	90 (3.62)	0.35 (0.19 - 0.63)	**<0.001**	0.53 (0.28 - 1.01)	
Conversion (%)							
No	298 (90.9)	70	80 (2.58)				
Yes	30 (9.1)	5	87 (7.22)	0.64 (0.26 - 1.6)	0.344		
Tumor location (%)							
Colon	206 (62.8)	49	80 (3.05)				
Rectum	122 (37.2)	26	83 (3.88)	0.91 (0.57 - 1.47)	0.709		
Procedure (%)							
Anterior rectum resection	102 (31.1)	21	83 (4.18)				
Left hemicolectomy	81 (24.7)	20	81 (4.62)	1.11 (0.6 - 2.05)	0.742		
Rectum extirpation	20 (6.1)	5	78 (10.35)	1.32 (0.5 - 3.5)	0.576		
Right hemicolectomy	125 (38.1)	29	80 (4.02)	1.18 (0.67 - 2.07)	0.568		
pN (%)							
N0	217 (66.2)	39	86 (2.77)				**0.003**
N+	111 (33.8)	36	71 (4.7)	1.86 (1.18 - 2.94)	**0.007**	2.01 (1.27 - 3.17)	
Resected lymph nodes (%)							
<20	161 (49.1)	41	78 (3.58)				
≥20	167 (50.9)	34	83 (3.29)	0.76 (0.48 - 1.2)	0.241		
**LNY (continuous, per lymph node)**	328 (100)	75	–	0.98 (0.96 - 1.01)	0.175		
L-status (%)							
L0	230 (70.1)	47	83 (2.84)				
L1	98 (29.9)	28	77 (4.47)	1.42 (0.89 - 2.26)	0.145		
V-status (%)							
V0	304 (92.7)	66	82 (2.5)				
V1	24 (7.3)	9	69 (9.2)	1.75 (0.87 - 3.52)	0.114		
Neoadjuvant chemotherapy (%)							
No	295 (89.9)	72	80 (2.58)				
Yes	33 (10.1)	3	96 (4.96)	0.38 (0.12 - 1.2)	0.098		
Neoadjuvant radiotherapy (%)							
No	290 (88.4)	69	80 (2.59)				
Yes	38 (11.6)	6	88 (6.5)	0.69 (0.3 - 1.58)	0.377		

Data are presented as n (%). Bold type indicates *p*-values < 0.05. LNY is presented as median [interquartile range]: 20 [16, 27]. The multivariable analysis included only variables significant in the univariable analysis. The multivariable Cox regression model was stratified by the Charlson Comorbidity Index because the proportional hazards assumption was violated for this variable. Therefore, no adjusted hazard ratio or p-value was estimated for the Charlson Comorbidity Index. For the regression analyses, complete-case analysis was performed, resulting in the inclusion of 328 of 333 patients (98.5%).

In the univariable analysis, laparoscopic surgery (*p* = 0.041), robotic surgery (*p* < 0.001), elective surgery (*p* = 0.040), N0 status (*p* = 0.007), and a lower Charlson Comorbidity Index (*p* < 0.001) were significantly associated with better overall survival.

In the multivariable analysis, nodal stage (*p* = 0.003) remained an independent predictor of overall survival.

Age, sex, conversion, tumor location, surgical procedure, neoadjuvant chemotherapy and neoadjuvant radiotherapy were not associated with overall survival.

### 3.5 Univariable and multivariable regression analyses for DFS

The findings of the univariable and multivariable Cox regression analyses for disease-free survival are shown in **Table 3**. In univariable analysis, colon cancer (*p* = 0.040), right hemicolectomy (*p* = 0.044), neoadjuvant chemotherapy (*p* = 0.001), and neoadjuvant radiotherapy (*p* = 0.003) were associated with better disease-free survival.

**Table 3:** Univariable and multivariable Cox regression analyses of disease-free survival.

Characteristics	All patients (n=306)	Events (n=42)		Univariate analysis		Multivariate analysis	
			Restricted mean survival time in months (SE)	Hazard ratio (95% CI)	*P*-value	Hazard ratio (95% CI)	*P*-value
Age (%)							
<50	51 (16.7)	10	84 (6)				
≥50	255 (83.3)	32	89 (2.63)	0.69 (0.34 - 1.42)	0.316		
Gender (%)							
Female	123 (40.2)	14	92 (3.28)				
Male	183 (59.8)	28	86 (3.28)	1.39 (0.73 - 2.64)	0.314		
Charlson comorbidity index (%)							
<3	116 (37.9)	21	86 (3.84)				
≥3	190 (62.1)	21	90 (3.02)	0.71 (0.39 - 1.3)	0.261		
Urgency of surgery (%)							
Elective	268 (87.6)	37	88 (2.58)				
Emergency	38 (12.4)	5	88 (6.98)	1.19 (0.47 - 3.02)	0.72		
Surgical approach (%)							
Open	103 (33.7)	15	86 (4.41)				
Laparoscopic	93 (30.4)	11	92 (3.57)	0.64 (0.29 - 1.39)	0.26		
Robotic	110 (35.9)	16	86 (4.35)	0.85 (0.42 - 1.73)	0.661		
Conversion (%)							
No	281 (91.8)	38	88 (2.55)				
Yes	25 (8.2)	4	88 (7.89)	1.04 (0.37 - 2.91)	0.941		
Tumor location (%)							
Colon	192 (62.7)	21	92 (2.59)				0.376
Rectum	114 (37.3)	21	81 (4.52)	1.89 (1.03 - 3.46)	**0.04**	1.4 (0.67 - 2.91)	
Procedure (%)							
Anterior rectum resection	94 (30.7)	16	83 (4.79)				
Left hemicolectomy	76 (24.8)	12	88 (4.4)	0.81 (0.38 - 1.71)	0.574		
Rectum extirpation	20 (6.5)	5	73 (11.92)	1.7 (0.62 - 4.64)	0.304		
Right hemicolectomy	116 (37.9)	9	96 (2.78)	0.43 (0.19 - 0.98)	**0.044**		
pN (%)							
N0	206 (67.3)	17	95 (2.4)				**0.011**
N+	100 (32.7)	25	75 (5.22)	3.43 (1.84 - 6.39)	**<0.001**	2.58 (1.24 - 5.36)	
Resected lymph nodes (%)							
<20	148 (48.4)	18	89 (3.62)				
≥20	158 (51.6)	24	88 (3.21)	1.17 (0.64 - 2.16)	0.605		
**LNY (continuous, per lymph node)**	306 (100)	42	–	1.00 (0.97 - 1.03)	0.904		
L-status (%)							
L0	217 (70.9)	24	91 (2.67)				0.713
L1	89 (29.1)	18	81 (4.81)	1.98 (1.07 - 3.65)	**0.029**	1.15 (0.54 - 2.45)	
V-status (%)							
V0	285 (93.1)	33	90 (2.41)				**0.012**
V1	21 (6.9)	9	61 (10.49)	4.18 (2 - 8.74)	**<0.001**	3.07 (1.37 - 6.85)	
Neoadjuvant chemotherapy (%)							
No	274 (89.5)	32	90 (2.43)				0.292
Yes	32 (10.5)	10	67 (10.15)	3.24 (1.58 - 6.64)	**0.001**	4.42 (0.23 - 84.98)	
Neoadjuvant radiotherapy (%)							
No	270 (88.2)	32	90 (2.44)				0.749
Yes	36 (11.8)	10	71 (9.29)	2.94 (1.43 - 6.01)	**0.003**	0.62 (0.03 - 11.84)	

Data are presented as n (%). Bold type indicates *p*-values < 0.05. LNY is presented as median [interquartile range]: 20 [16, 28]. The multivariable analysis included only variables significant in the univariable analysis. For the regression analyses, complete-case analysis was performed, resulting in the inclusion of 306 of 333 patients (91.9%).

Among histopathological parameters, lymph node status (*p* < 0.001), lymphatic invasion (*p* = 0.029), and venous invasion (*p* < 0.001) were significantly associated with disease-free survival in univariable analysis, whereas only lymphatic invasion (*p* = 0.011) and venous invasion (*p* = 0.012) were confirmed as independent predictors of disease-free survival in the multivariable model.

Consistent with the Kaplan-Meier analysis, higher LNY was not associated with DFS (*p* = 0.605). When analyzed as a continuous variable, LNY was likewise not significantly associated with DFS (HR per additional lymph node, 1.00; 95% CI, 0.97 - 1.03; *p* = 0.904). Age at surgery, sex, Charlson Comorbidity Index, urgency of surgery, surgical approach, and conversion to open surgery were also not significantly associated with DFS.

## 4 Discussion

In our retrospective single-center cohort of patients with stage I–III colorectal cancer undergoing curative-intent resection, LNY ≥20 was not significantly associated with overall or disease-free survival in the overall cohort. However, in procedure-specific exploratory analyses, LNY ≥20 was associated with improved overall survival in patients undergoing anterior rectal resection. Given the exploratory nature of the subgroup analyses and the absence of formal adjustment for multiple comparisons, the observed association between LNY ≥20 and improved OS after anterior rectal resection should be interpreted with caution. In addition, higher LNY was linked to distinct clinicopathological features, including tumor location, advanced local tumor stage, microsatellite instability, and the neoadjuvant treatment.

Current guidelines recommend the examination of at least 12 lymph nodes for adequate staging in CRC (8–10). Nevertheless, there is still no final consensus in the literature regarding the optimal number of lymph nodes required to maximize staging accuracy and survival prediction, with proposed thresholds ranging from 7 to 30 nodes depending on study population and methodology (8, 9). In line with our data, Del Paggio et al. reported that in stage II colon cancer, no additional survival benefit was observed beyond a cut-off of 20 lymph nodes, whereas no upper threshold could be identified in stage III disease (21). In contrast, Foo et al. found that a significant improvement in DFS and OS was only observed in patients with LNY ≥20, while no significant difference was seen between patients with <12 and 12–19 lymph nodes (31). In their multivariable analysis, LNY ≥20 remained an independent predictor of favorable DFS (31). Further examples of studies analyzing the association between LNY and patient outcome include an analysis of the National Cancer Database (NCDB) including 261,423 patients, which showed that LNY of ≥24 nodes was associated with improved overall survival across all N-stages (20). Similarly, a recent NCDB study including 195,009 patients identified ≥18 nodes as a superior threshold compared with the current standard of 12 nodes, showing improved overall survival (26). A 20-year study from New Zealand reported the strongest association at 12 nodes, whereas no significant additional survival differences were observed beyond 22 nodes (19).

A key finding of our procedure-specific exploratory analysis was that LNY ≥20 was associated with improved overall survival in patients undergoing anterior rectal resection, whereas no comparable survival benefit was observed after left- or right-sided hemicolectomy. This finding is particularly relevant because the prognostic value of LNY in rectal cancer remains controversial, especially in the context of neoadjuvant treatment. In our cohort, neoadjuvant chemotherapy and neoadjuvant radiotherapy were significantly associated with LNY <20. This is consistent with previous evidence showing that neoadjuvant treatment reduces the number of retrievable lymph nodes. A systematic review and meta-analysis including 34 studies and 37 datasets quantified this effect, reporting a mean reduction of 3.9 lymph nodes after neoadjuvant chemoradiotherapy and 2.1 lymph nodes after neoadjuvant radiotherapy alone (39). Another study including 615 patients confirmed an approximately 33% reduction in LNY after neoadjuvant treatment (40). As it is not expected that the surgical radicality of total mesorectal excision varies depending on the use of neoadjuvant treatment, the lower LNY may reflect a treatment-related reduction in pathological lymph node detectability rather than differences in the extent of surgical resection. Despite this treatment-related reduction in LNY, some studies suggest that lymph node yield may still retain prognostic relevance after neoadjuvant chemoradiotherapy. One recent study including 1,596 patients identified LNY <12 as an independent risk factor for both overall survival and disease-free survival after neoadjuvant chemoradiotherapy (41). Likewise, an NCDB analysis reported that LNY ≥12 was associated with improved survival even after neoadjuvant radiotherapy (42). In contrast, the PROCARE study found that lymph node count did not provide additional prognostic information after chemoradiotherapy, whereas in patients treated with surgery alone, each additional lymph node was associated with a 2.7% reduction in the risk of death (43). The association of a higher lymph node yield with improved overall survival, but not disease-free survival, in patients undergoing anterior resection suggests that its prognostic benefit may not be explained solely by improved recurrence control.

In line with previous studies, LNY in our cohort was strongly associated with primary tumor localization. Patients with colon cancer were more likely to have LNY ≥20 in comparison to patients with rectal cancer. This observation is consistent with previous reports showing that right-sided and colonic tumors generally yield higher numbers of lymph nodes than left-sided or rectal tumors. Betge et al. reported a median LNY of 20 for right-sided tumors, 13 for left-sided tumors, and 15 for rectal tumors, with right-sided location being an independent predictor of higher LNY (44). These findings support the interpretation that LNY reflects not only surgical and pathological quality, but also anatomical and tumor-related factors.

Beyond anatomical factors, our data suggest that LNY is also influenced by clinicopathological and biological tumor characteristics. In our cohort, higher LNY was associated with more advanced local tumor stage and microsatellite instability. This is consistent with previous studies showing increased LNY in tumors with higher T stage and in microsatellite instability-high (MSI-H) or deficient mismatch repair (dMMR) CRC. Bækgaard et al. reported a significantly higher median LNY in dMMR tumors compared with proficient mismatch repair (pMMR) tumors in a retrospective cohort study including 9,705 patients with stage I–III colon cancer (25). Similarly, Sun et al. confirmed in a cohort of 4,205 patients that MSI-H tumors had a significantly higher median LNY than microsatellite stable (MSS) and microsatellite instability-low (MSI-L) tumors, and that MSI-H status was an independent predictor of increased LNY in colon cancer (45). Furthermore, Betge et al. found that LNY was independently associated with T-stage, N-stage, tumor size, and right-sided location (44). Ahmadi et al., analyzing 14,646 patients, showed that younger age, right-sided disease, higher T-stage, female sex, and the absence of neoadjuvant radiotherapy in rectal cancer were associated with higher LNY (46).

The present study has several limitations that should be considered when interpreting the findings. First, the retrospective single-center design may have introduced selection bias and residual confounding due to baseline differences between the study groups, thereby limiting the generalizability of the findings. Second, LNY was dichotomized using a cutoff of 20 lymph nodes, although the optimal threshold remains uncertain and may vary according to tumor location, disease stage, and treatment strategy. Third, the findings of the present study should be interpreted in light of the cohort size and limited statistical power, particularly regarding stage-specific and procedure-specific subgroup analyses. The subgroup analyses, particularly the procedure-specific analysis of patients undergoing anterior rectal resection, were exploratory and should therefore be interpreted with caution. As neoadjuvant therapy is a key determinant of LNY, a potential confounding effect on the observed association between LNY and survival cannot be excluded. Fourth, additional pathological prognostic parameters, including tumor budding, circumferential resection margin status in rectal cancer, tumor regression grade after neoadjuvant therapy, and tumor deposits (N1c), were not consistently available and therefore could not be included in the present analysis. Finally, LNY is influenced not only by surgical quality, but also by pathological processing, anatomical factors and tumor biology.

In this retrospective single-center study of stage I–III colorectal cancer, retrieval of 20 or more lymph nodes was associated with distinct tumor- and treatment-related characteristics but not with improved disease-free survival or overall survival in the overall cohort. Only the subgroup of patients undergoing anterior rectal resection showed an association between higher lymph node yield and improved overall survival, without a corresponding benefit in disease-free survival. These findings suggest that lymph node yield should be interpreted primarily as a marker of staging adequacy and case-specific context rather than as an independent therapeutic target. Overall, our findings suggest that a higher lymph node yield is not necessarily associated with improved oncological outcomes but may be relevant in selected clinical contexts, warranting further investigation.

## Data Availability

The raw data supporting the conclusions of this article will be made available by the authors, without undue reservation.

## Supplementary material

Supplementary Figure 1 Kaplan-Meier curves showing the association between lymph node yield and overall survival **(A, C)** and disease-free survival **(B, D)** according to lymphatic invasion status, with L0 status shown in **(A, B)** and L1 status in **(C, D)**.

## References

[1] Bray F Laversanne M Sung H Ferlay J Siegel RL Soerjomataram I . Global cancer statistics 2022: GLOBOCAN estimates of incidence and mortality worldwide for 36 cancers in 185 countries. CA Cancer J Clin. (2024) 74:229–63. doi: 10.3322/caac.21834 38572751

[2] Collaborators GBDC . The global, regional, and national burden of cancer, 1990-2023, with forecasts to 2050: a systematic analysis for the Global Burden of Disease Study 2023. Lancet. (2025) 406:1565–86. doi: 10.1016/S0140-6736(25)01635-6 PMC12687902 41015051

[3] Argiles G Tabernero J Labianca R Hochhauser D Salazar R Iveson T . Localised colon cancer: ESMO Clinical Practice Guidelines for diagnosis, treatment and follow-up. Ann Oncol. (2020) 31:1291–305. doi: 10.1016/j.annonc.2020.06.022 32702383

[4] Glynne-Jones R Wyrwicz L Tiret E Brown G Rodel C Cervantes A . Rectal cancer: ESMO Clinical Practice Guidelines for diagnosis, treatment and follow-up. Ann Oncol. (2017) 28:iv22–40. doi: 10.1093/annonc/mdx224 28881920

[5] Vogel JD Felder SI Bhama AR Hawkins AT Langenfeld SJ Shaffer VO . The american society of colon and rectal surgeons clinical practice guidelines for the management of colon cancer. Dis Colon Rectum. (2022) 65:148–77. doi: 10.1097/dcr.0000000000002323 34775402

[6] Resch A Langner C . Lymph node staging in colorectal cancer: old controversies and recent advances. World J Gastroenterol. (2013) 19:8515–26. doi: 10.3748/wjg.v19.i46.8515 24379568 PMC3870496

[7] Parsons HM Tuttle TM Kuntz KM Begun JW McGovern PM Virnig BA . Association between lymph node evaluation for colon cancer and node positivity over the past 20 years. JAMA. (2011) 306:1089–97. doi: 10.1001/jama.2011.1285 21917579

[8] National Comprehensive Cancer Network . Colon Cancer 2026 (2026). Available online at: https://www.nccn.org/professionals/physician_gls/pdf/colon.pdf (Accessed April 21, 2026).

[9] National Comprehensive Cancer Network . Rectal Cancer 2026 (2026). Available online at: https://www.nccn.org/professionals/physician_gls/pdf/rectal.pdf (Accessed April 21, 2026).

[10] Amin MB Greene FL Edge SB Compton CC Gershenwald JE Brookland RK . Ajcc Cancer Staging Manual. 8th ed. New York: Springer (2017). 10.3322/caac.21388 28094848

[11] Chang GJ Rodriguez-Bigas MA Skibber JM Moyer VA . Lymph node evaluation and survival after curative resection of colon cancer: systematic review. J Natl Cancer Inst. (2007) 99:433–41. doi: 10.1093/jnci/djk092 17374833

[12] Wilkie BD Chan JM Paratz E Proud D Smart P An V . Lymph node yield and colorectal cancer survival: A bowel cancer outcomes registry cohort study. Anz J Surg. (2025) 95:1509–16. doi: 10.1111/ans.70150 40265735

[13] Lykke J Rosenberg J Jess P Roikjaer O Danish Colorectal Cancer G . Lymph node yield and tumour subsite are associated with survival in stage I-III colon cancer: results from a national cohort study. World J Surg Oncol. (2019) 17:62. doi: 10.1186/s12957-019-1604-x 30940175 PMC6446268

[14] Lu J Xing J Zang L Zhang C Xu L Zhang G . Extent of lymphadenectomy for surgical management of right-sided colon cancer: the randomized phase III RELARC trial. J Clin Oncol. (2024) 42:3957–66. doi: 10.1200/jco.24.00393 39190853

[15] Li K Li H Wu A Zang L Zhang G Xu L . Long-term survival on extent of lymphadenectomy for right-sided colon cancer: five-year follow-up results of a randomized controlled trial (RELARC trial). Ann Surg. (2025). doi: 10.1097/sla.0000000000006941 40938728

[16] Georgiou P Tan E Gouvas N Antoniou A Brown G Nicholls RJ . Extended lymphadenectomy versus conventional surgery for rectal cancer: a meta-analysis. Lancet Oncol. (2009) 10:1053–62. doi: 10.1016/s1470-2045(09)70224-4 19767239

[17] Kojima T Hino H Shiomi A Kagawa H Yamaoka Y Manabe S . Long-term outcomes of D2 vs. D3 lymph node dissection for cT2N0M0 colorectal cancer: a multi-institutional retrospective analysis. Int J Clin Oncol. (2022) 27:1717–24. doi: 10.1007/s10147-022-02236-3 36029376

[18] Ouchi A Komori K Kimura K Kinoshita T Shimizu Y Nagino M . Survival benefit of Japanese extended lymphadenectomy for clinically node-negative and node-positive colorectal cancers. Dis Colon Rectum. (2018) 61:162–71. doi: 10.1097/dcr.0000000000000957 29337770

[19] Men V Bahl P Jin JZ Singh PP Hill AG . Lymph node yield and long-term mortality risk in patients with colon cancer: A 20-year follow-up national study. Ann Surg Oncol. (2025) 32:1117–27. doi: 10.1245/s10434-024-16428-w 39496903

[20] Trepanier M Erkan A Kouyoumdjian A Nassif G Albert M Monson J . Examining the relationship between lymph node harvest and survival in patients undergoing colectomy for colon adenocarcinoma. Surgery. (2019) 166:639–47. doi: 10.1016/j.surg.2019.03.027 31399220

[21] Del Paggio JC Peng Y Wei X Nanji S MacDonald PH Krishnan Nair C . Population-based study to re-evaluate optimal lymph node yield in colonic cancer. Br J Surg. (2017) 104:1087–96. doi: 10.1002/bjs.10540 28542954 PMC7938819

[22] Bundred J Lal N Chan DKH Buczacki SJA . Lymph node yield as a surrogate marker for tumour biology and prognosis in colon cancer. Br J Cancer. (2025) 132:643–51. doi: 10.1038/s41416-025-02949-y 39953281 PMC11961567

[23] Lal N Chan DKH Ng ME Vermeulen L Buczacki SJA . Primary tumour immune response and lymph node yields in colon cancer. Br J Cancer. (2022) 126:1178–85. doi: 10.1038/s41416-022-01700-1 35043009 PMC9023574

[24] Lee SH Pankaj A Neyaz A Ono Y Rickelt S Ferrone C . Immune microenvironment and lymph node yield in colorectal cancer. Br J Cancer. (2023) 129:917–24. doi: 10.1038/s41416-023-02372-1 37507544 PMC10491581

[25] Baekgaard FW Iordache A Nordholm AH Krarup PM Schlesinger NH Qvortrup C . The prognostic impact of lymph node yield in deficient and proficient mismatch repair colon cancers: A retrospective national cohort study. Colorectal Dis. (2025) 27:e70332. doi: 10.1111/codi.70332 41339659

[26] Chatani PD Fredette JD Sun J Thomas RP Porpiglia AS Greco SH . Redefining the lymphadenectomy standard to avoid understaging in oncologic colectomies. J Natl Compr Canc Netw. (2026) 24:1–8. doi: 10.6004/jnccn.2025.7122 41911898

[27] Liu T Jiao S Gao S Shi Y . Optimal lymph node yield for long-term survival in elderly patients with right-sided colon cancer: a large population-based cohort study. BMC Cancer. (2025) 25:590. doi: 10.1186/s12885-025-13987-3 40170177 PMC11963392

[28] Sun X Li R Zhao W Wang S Liu H Gao W . Optimal lymph node count for colorectal cancer surgery: A cohort study utilizing real-world data. Surg Laparosc Endosc Percutan Tech. (2026) 36:e1441. doi: 10.1097/sle.0000000000001441 41575773

[29] Xiang R Yang B Sun J Fu J Wu J Wang J . Effect of examined lymph node count on precise cancer-specific survival and effectiveness of adjuvant chemotherapy in resected colorectal cancer. Sci Rep. (2025) 15:25856. doi: 10.1038/s41598-025-03999-1 40670403 PMC12267557

[30] Tian Y Qiao X Zheng G Dan H Dou X Ren G . Optimal number of the examined lymph nodes for different N stages in colorectal cancer. Eur J Med Res. (2025) 30:753. doi: 10.1186/s40001-025-02990-w 40813992 PMC12351765

[31] Foo CC Ku C Wei R Yip J Tsang J Chan TY . How does lymph node yield affect survival outcomes of stage I and II colon cancer? World J Surg Oncol. (2020) 18:22. doi: 10.1186/s12957-020-1802-6 31996214 PMC6990535

[32] Simoes P Fernandes G Costeira B Machete M Baptista C D NS . Lymph node yield in the pathological staging of resected nonmetastatic colon cancer: The more the better? Surg Oncol. (2022) 43:101806. doi: 10.1016/j.suronc.2022.101806 35841744

[33] von Elm E Altman DG Egger M Pocock SJ Gotzsche PC Vandenbroucke JP . The Strengthening the Reporting of Observational Studies in Epidemiology (STROBE) statement: guidelines for reporting observational studies. PloS Med. (2007) 4:e296. doi: 10.1136/bmj.39335.541782.ad 17941714 PMC2020495

[34] Harris PA Taylor R Thielke R Payne J Gonzalez N Conde JG . Research electronic data capture (REDCap)--a metadata-driven methodology and workflow process for providing translational research informatics support. J BioMed Inform. (2009) 42:377–81. doi: 10.1016/j.jbi.2008.08.010 18929686 PMC2700030

[35] Harris PA Taylor R Minor BL Elliott V Fernandez M O'Neal L . The REDCap consortium: Building an international community of software platform partners. J BioMed Inform. (2019) 95:103208. doi: 10.1016/j.jbi.2019.103208 31078660 PMC7254481

[36] Quan H Li B Couris CM Fushimi K Graham P Hider P . Updating and validating the Charlson comorbidity index and score for risk adjustment in hospital discharge abstracts using data from 6 countries. Am J Epidemiol. (2011) 173:676–82. doi: 10.1093/aje/kwq433 21330339

[37] Dindo D Demartines N Clavien PA . Classification of surgical complications: a new proposal with evaluation in a cohort of 6336 patients and results of a survey. Ann Surg. (2004) 240:205–13. doi: 10.1097/01.sla.0000133083.54934.ae PMC1360123 15273542

[38] Schmiegel W Buchberger B Follmann M Graeven U Heinemann V Langer T . Not available. Z Gastroenterol. (2017) 55:1344–498. 10.1055/s-0043-121106 29212104

[39] Mechera R Schuster T Rosenberg R Speich B . Lymph node yield after rectal resection in patients treated with neoadjuvant radiation for rectal cancer: A systematic review and meta-analysis. Eur J Cancer. (2017) 72:84–94. doi: 10.1016/j.ejca.2016.10.031 28027520

[40] Ha YH Jeong SY Lim SB Choi HS Hong YS Chang HJ . Influence of preoperative chemoradiotherapy on the number of lymph nodes retrieved in rectal cancer. Ann Surg. (2010) 252:336–40. doi: 10.1097/sla.0b013e3181e61e33 20647928

[41] Jo MH Kim DW Lee JA Choi MJ Shin HR Lee TG . The impact of lymph node harvest on recurrence in rectal cancer following neoadjuvant chemoradiotherapy. J Gastrointest Surg. (2026) 30:102447. doi: 10.1016/j.gassur.2026.102447 42066946

[42] Cox ML Adam MA Shenoi MM Turner MC Sun Z Mantyh CR . Resected irradiated rectal cancers: Are twelve lymph nodes really necessary in the era of neoadjuvant therapy? Am J Surg. (2018) 216:444–9. doi: 10.1016/j.amjsurg.2017.08.014 28890055 PMC9757024

[43] Ceelen W Willaert W Varewyck M Libbrecht S Goetghebeur E Pattyn P . Effect of neoadjuvant radiation dose and schedule on nodal count and its prognostic impact in stage II-III rectal cancer. Ann Surg Oncol. (2016) 23:3899–906. doi: 10.1245/s10434-016-5363-4 27380639

[44] Betge J Harbaum L Pollheimer MJ Lindtner RA Kornprat P Ebert MP . Lymph node retrieval in colorectal cancer: determining factors and prognostic significance. Int J Colorectal Dis. (2017) 32:991–8. doi: 10.1007/s00384-017-2778-8 28210855 PMC5486641

[45] Sun X Li R Wang S Zhao W Liu H Gao W . Microsatellite instability-high status enhances lymph node yield and reduces optimal dissection thresholds in colorectal cancer: A retrospective cohort analysis with beta-binomial modeling. Surgery. (2026) 190:109890. doi: 10.1016/j.surg.2025.109890 41289775

[46] Ahmadi O Stringer MD Black MA McCall JL . Clinico-pathological factors influencing lymph node yield in colorectal cancer and impact on survival: analysis of New Zealand Cancer Registry data. J Surg Oncol. (2015) 111:451–8. doi: 10.1002/jso.23848 25663298

