# Supplementary material for: More is not always better: higher lymph node yield is not associated with improved survival in stage I–III colorectal cancer
Source: Front Oncol. 2026 Aug 19;16:1900616. doi: 10.3389/fonc.2026.1900616 (PMC13533714; doi:10.3389/fonc.2026.1900616)
Supplement: Supplementary file 1 [file Table1.docx]

**Supplementary Table 1. Median Follow-Up Times for Overall Survival and Disease-Free Survival**

| **Endpoint** | **Patients (n)** | **Events (n)** | **Median Follow-Up (Months)** | **95% CI** |
| --- | --- | --- | --- | --- |
| Overall Survival | 332 | 75 | 49.6 | 47.7 - 52.1 |
| Disease-Free Survival | 310 | 42 | 45.9 | 41.2 - 49.6 |
